# Isolation and Characterization of Poecistasin, an Anti-Thrombotic Antistasin-Type Serine Protease Inhibitor from Leech *Poecilobdella manillensis*

**DOI:** 10.3390/toxins10110429

**Published:** 2018-10-26

**Authors:** Xiaopeng Tang, Mengrou Chen, Zilei Duan, James Mwangi, Pengpeng Li, Ren Lai

**Affiliations:** 1 Key Laboratory of Animal Models and Human Disease Mechanisms of Chinese Academy of Sciences/Key Laboratory of Bioactive Peptides of Yunnan Province, Kunming Institute of Zoology, Kunming 650223, Yunnan, China; tangxiaopeng@mail.kiz.ac.cn (X.T.); duanzilei@mail.kiz.ac.cn (Z.D.); mwangij1124@yahoo.com (J.M.); 2 Kunming College of Life Science, University of Chinese Academy of Sciences, Kunming 650204, Yunnan, China; 3 College of Life Sciences, Nanjing Agricultural University, Nanjing 210095, Jiangsu, China; chenmengrou@outlook.com (M.C.); pengpengli59@gmail.com (P.L.)

**Keywords:** Antistasin-type inhibitor, *Poecilobdella manillensis*, poecistasin, coagulation, thrombus formation, ischemic stroke

## Abstract

Antistasin, first identified as a potent inhibitor of the blood coagulation factor Xa, is a novel family of serine protease inhibitors. In this study, we purified a novel antistasin-type inhibitor from leech *Poecilobdella manillensis* called poecistasin. Amino acid sequencing of this 48-amino-acid protein revealed that poecistasin was an antistasin-type inhibitor known to consist of only one domain. Poecistasin inhibited factor XIIa, kallikrein, trypsin, and elastase, but had no inhibitory effect on factor Xa and thrombin. Poecistasin showed anticoagulant activities. It prolonged the activated partial thromboplastin time and inhibited FeCl_3_-induced carotid artery thrombus formation, implying its potent function in helping *Poecilobdella manillensis* to take a blood meal from the host by inhibiting coagulation. Poecistasin also suppressed ischemic stroke symptoms in transient middle cerebral artery occlusion mice model. Our results suggest that poecistasin from the leech *Poecilobdella manillensis* plays a crucial role in blood-sucking and may be an excellent candidate for the development of clinical anti-thrombosis and anti-ischemic stroke medicines.

## 1. Introduction

Protease inhibitors play important roles in the biological purposes of venomous animals, for example, predation and defense [1,2]. There are nine classes of proteases already reported in previous studies, including named molecules (Aspartic, Cysteine, Glutamic, Metallo, Asparagine, Serine and Threonine), unknown molecules (Unknown) and molecules of mixed catalytic mechanisms (Mixed) [3,4]. For each protease class, inhibitors are already described [3,4]. Serine protease inhibitors represent a diverse class of proteins that have been subdivided into many distinct families [5], such as the Kazal [6,7,8,9], Kunitz [10,11,12], Bowman-Birk [13,14,15], SSI [16,17,18,19] and Chelonianin [20,21] families. Antistasin is another kind of serine protease inhibitor that contains cysteine-rich 119-amino-acid protein isolated from Mexican leech *Haementeria officinalis* [22]. Antistasin showed no close sequence similarity to other known protease inhibitors and thus became the prototype of a novel family [23,24].

Antistasin-type inhibitors were found in many living organisms [25,26,27] and several antistasin-type inhibitors were isolated from leeches [22,28,29,30,31,32,33]. An anticoagulant antistasin-type inhibitor named ghilanten was isolated from the salivary glands of south American leech *Haementeria ghilianii* [28]. Ghilanten prolonged prothrombin time by inhibiting the factor Xa [28]. Hirustasin was purified from leech *Hirudo medicinalis* and was the first inhibitor of tissue kallikrein without inhibitory effect on factor Xa (FXa) [29]. Hirustasin was the first family member comprising only one antistasin-like domain [29]. Bdellastasin is another antistasin-type inhibitor from leech *Hirudo medicinalis* [30]. Bdellastasin inhibited bovine trypsin and human plasmin but had no inhibitory effect on FXa, thrombin, tissue kallikrein, plasma kallikrein and chymotrypsin [30]. An antistasin-type protease inhibitor named as piguamerin from Korean leech *Hirudo nipponia* potently inhibited plasma and tissue kallikreins and trypsin [31]. Antistasin-type protease inhibitors guamerin and guamerin II with elastase inhibitory effect have been isolated from blood-sucking leech *Hirudo nipponia* and non-blood-sucking leech *Whitmania edentula* extracts, respectively [32,33].

Here, we purified and characterized an antistasin-type serine protease inhibitor from leech of *Poecilobdella manillensis* (*P. manillensis*), called poecistasin. Poecistasin comprises one antistasin-like domain and is the first antistasin-type inhibitor from leech of *P. manillensis*. Poecistasin inhibited factor XIIa (FXIIa), kallikrein, trypsin and elastase, but had no inhibitory effect on FXa and thrombin. Poecistasin showed an anticoagulant activity by inhibiting both FXIIa and kallikrein of the intrinsic coagulation pathway. This implies its potent function in helping *P. manillensis* to take a blood meal from the host.

## 2. Results

### 2.1. Purification of Poecistasin

**Figure 1:**
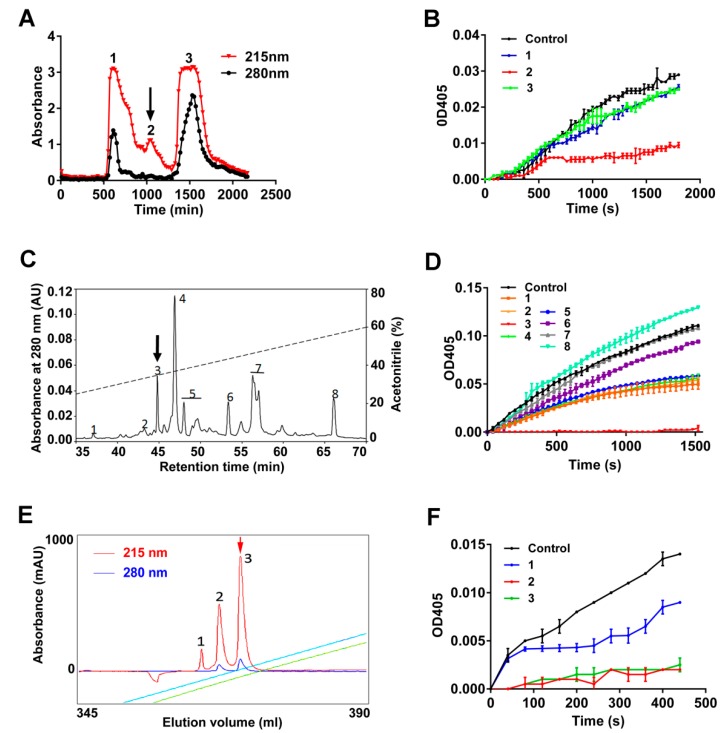
Purification of poecistasin from *P. manillensis*. (**A**) The secretions of *P. manillensis* were separated by Sephadex G-50 column by monitoring at both 215 and 280 nm. The fraction exerts FXIIa inhibitory activity is indicated by an arrow. (**B**) The fractions of “A” were used to test FXIIa inhibitory activity. (**C**) The fraction of previous step exerts FXIIa inhibitory activity was further purified by C_8_ RP-HPLC by monitoring at 280 nm. The protein peak exerts FXIIa inhibitory activity is indicated by an arrow. The dashed line represents a line gradient of acetonitrile from 30 to 60% over 35 min. (**D**) The peaks of “C” was used to test FXIIa inhibitory activity. (**E**) The peak of previous step exerts FXIIa inhibitory activity were further purified by a Mono S^TM^ 5/50 GL column connected to AKTA FPLC system by monitoring at both 215 and 280 nm. The blue and green line represents the conductivity and NaCl concentration, respectively. The protein peak that was used for liquid chromatography coupled with tandem mass spectrometry (LC−MS/MS) is indicated by a red arrow. (**F**) The peaks of “E” were used to test FXIIa inhibitory activity. Control means a lack of addition of any peak. (**B**,**D**,**F**) are representative of at least five independent experiments.

*P. manillensis* secretions were diluted in phosphate buffer (PB buffer) and three fractions were obtained in the chromatographic step using the Sephadex G-50 column (Figure 1A). The fraction which can inhibit FXIIa enzymatic activity (Figure 1B) is indicated by an arrow (Figure 1A). The fraction that inhibits the FXIIa activity was then subjected to a reverse-phase high performance liquid chromatography (RP-HPLC) using a C8 column (Figure 1C), and the peak with inhibitory activity on FXIIa, indicated by an arrow, was lyophilized (Figure 1C,D). Finally, we got the purified peptide with FXIIa inhibiting activity, indicated by an arrow, named as poecistasin by using a Mono S^TM^ 5/50 GL column connected to AKTA explorer 10S fast protein liquid chromatography (FPLC) system (Figure 1E,F).

### 2.2. Primary Structure of Poecistasin

**Figure 2:**
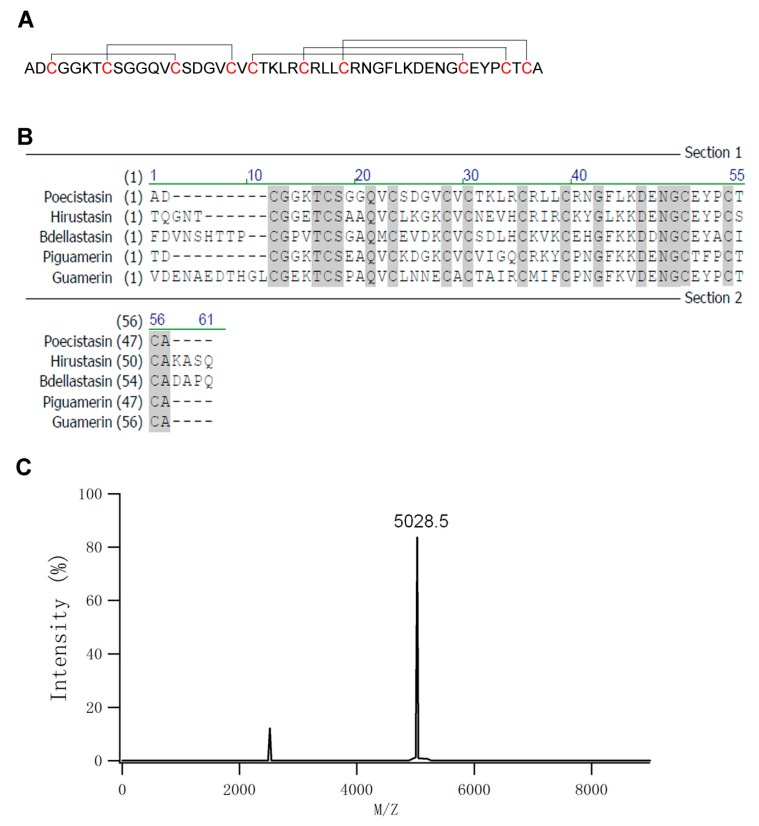
Primary structure of poecistasin. (**A**) Peptide sequence of poecistasin was determined by LC−MS/MS. The disulfide bridge pattern shown has been elucidated for Hirustasin before [30]. (**B**) Multiple sequence alignment of antistasin-type inhibitor Hirustasin (*Hirudo medicinalis*, P80302.1), Bdellastasin (*Hirudo medicinalis*, P82107.1), piguamerin (*Hirudo nipponia*, P81499.1) and Guamerin (*Hirudo nipponia*, P46443.1). Cysteines and conserved residues are indicated by shadowing. (**C**) MALDI-TOF analysis of purified native poecistasin.

The eluted peak 3 (Figure 1E) of FPLC containing FXIIa inhibitory activity was collected and lyophilized. Peptide sequence was determined by LC−MS/MS. The sequence of mature peptide of poecistasin (48 amino acids) is “ADCGGKTCSGGQVCSDGVCVCTKLRCRLLCRNGFLKDENGCEYPCTCA” (Figure 2A). Sequence alignment showed it was similar to hirustasin (*Hirudo medicinalis*; identity: 63%), Bdellastasin (*Hirudo medicinalis*; identity: 57%), piguamerin (*Hirudo nipponia*; identity: 69%) and guamerin (*Hirudo nipponia*; identity: 65%) which are antistasin-type serine protease inhibitors containing only one domain (Figure 2B). Matrix-assisted laser desorption ionization time-of-flight mass spectrometry (MALDI-TOF-MS) showed the molecular weight (MW) of native poecistasin is 5027.5 Da (Figure 2C).

### 2.3. Effects of Poecistasin on Proteases and Coagulation

**Figure 3:**
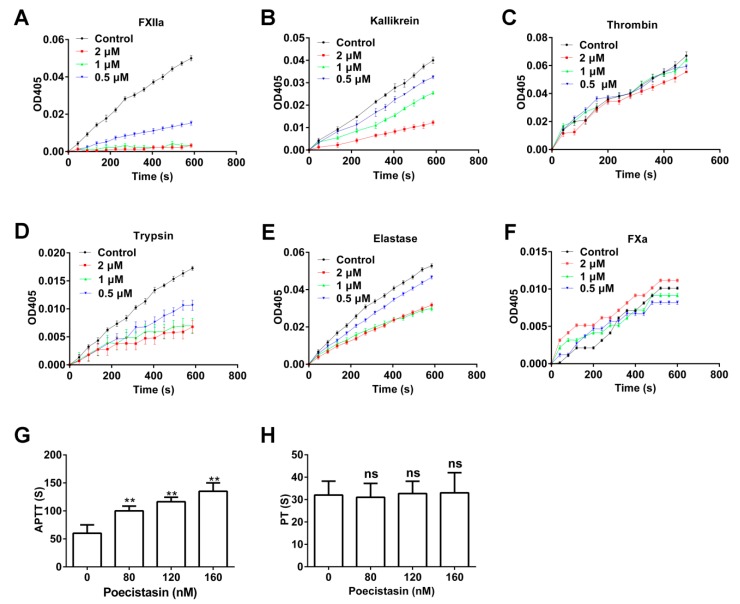
Effects of poecistasin on serine proteases and coagulation. (**A**–**F**) Effects of native poecistasin on FXIIa, kallikrein, thrombin, trypsin, elastase and FXa. (**G**,**H**) Effects of poecistasin on APTT and PT. (**A**–**H**) are representative of at least five independent experiments. Control means a lack of addition of poecistasin. Data represent mean ± SD, ** *p* < 0.01 by unpaired *t*-test. NS = no significance.

The activity of poecistasin on serine proteases (FXIIa, kallikrein, thrombin, trypsin, elastase and FXa) and coagulation (activated partial thromboplastin time (APTT) and prothrombin time (PT) assays) were investigated. As illustrated in Figure 3, native poecistasin showed strong inhibitory activity against FXIIa (Figure 3A), kallikrein (Figure 3B), trypsin (Figure 3D) and Elastase (Figure 3E) with *Kis* of 9.31, 51.97, 9.28 and 546.7 nM, respectively (Figure S1). However, native poecistasin showed no effect on thrombin and FXa (Figure 3C,F). Consistent with its strong inhibitory activities on FXIIa and kallikrein, native poecistasin prolonged APTT in a dose-dependent manner (Figure 3G) with no effect on PT (Figure 3H).

### 2.4. Effects of Poecistasin on FeCl_3_-Induced Carotid Artery Injury Model

**Figure 4:**
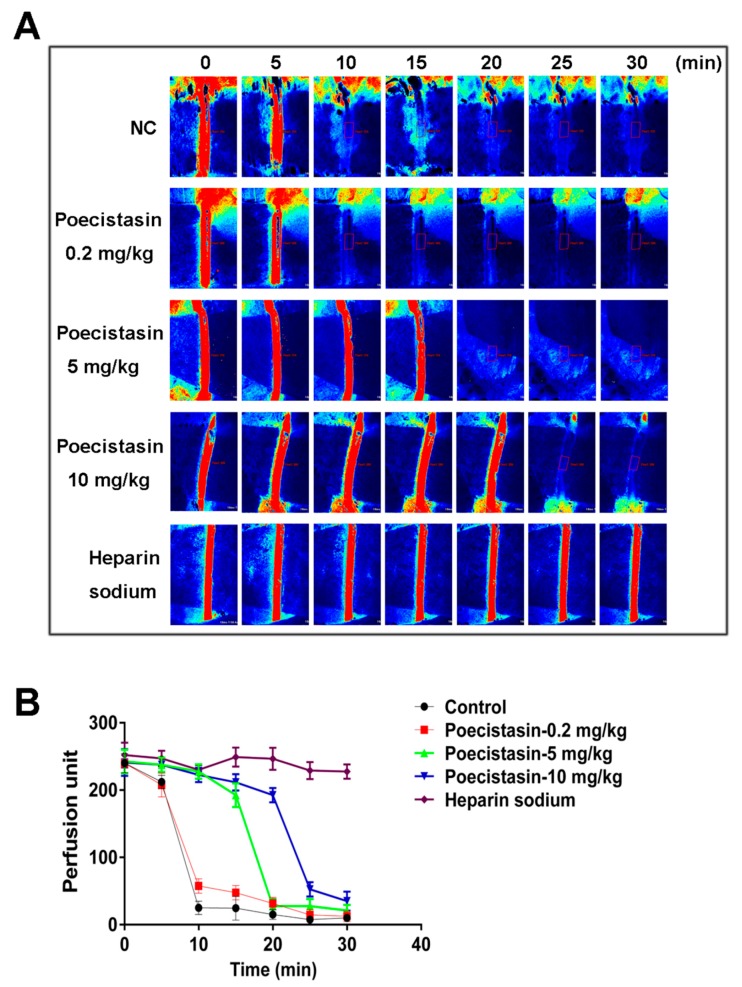
Effects of poecistasin on FeCl_3_-induced carotid artery injury model. Different concentrations of recombinant poecistasin (0.2, 5 and 10 mg/kg) and heparin sodium (20 mg/kg) were injected by tail vein 10 min before surgery. After treatment with 10% FeCl_3_, blood flow at region of interest (ROI, indicated by red box) was monitored for 0, 5, 10, 15, 20, 25 and 30 min, respectively. Representative images of carotid artery blood flow (**A**) by the laser speckle perfusion imaging and quantification of carotid artery blood flow (**B**) by measuring the perfusion unit of ROI are shown. NC: saline. Data represent mean ± SD (*n* = 6).

Recombinant poecistasin, which showed similar FXIIa inhibitory activity with native poecistasin, was expressed in *E. coli* and purified (Figure S2). As illustrated in Figure S2A, recombinant poecistasin was expressed induced by 1 mM isopropyl-β-d-thiogalactopyranoside (IPTG) for 6 h and the fusion poecistasin eluted from Ni^2+^ affinity chromatography column was cut by rTEV protease (Figure S2B). The fraction treated with rTEV protease was loaded onto a RP-HPLC C_8_ column to purify recombinant poecistasin (Figure S2C). As illustrated in Figure S2D, the four peaks from previous step were applied to test FXIIa enzymatic inhibitory activity and the “peak 4” with strong FXIIa enzymatic inhibitory activity was analyzed by MALDI-TOF-MS. MALDI-TOF-MS showed the MW of recombinant poecistasin is 5085.7 Da (Figure S2E). The effect of poecistasin on thrombosis in vivo was evaluated in carotid artery thrombus model induced by FeCl_3_ in C57BL/6J mice. Different concentrations of recombinant poecistasin (0.2, 5 and 10 mg/kg) and heparin sodium (20 mg/kg) were injected by tail vein 10 min before surgery. After the treatment with 10% FeCl_3_, blood flow was monitored for 0, 5, 10, 15, 20, 25 and 30 min, respectively. As shown in Figure 4A, B, poecistasin inhibited thrombus formation in a dose-dependent manner with a similar tendency to heparin sodium.

### 2.5. Effects of Poecistasin on Stroke Model

**Figure 5:**
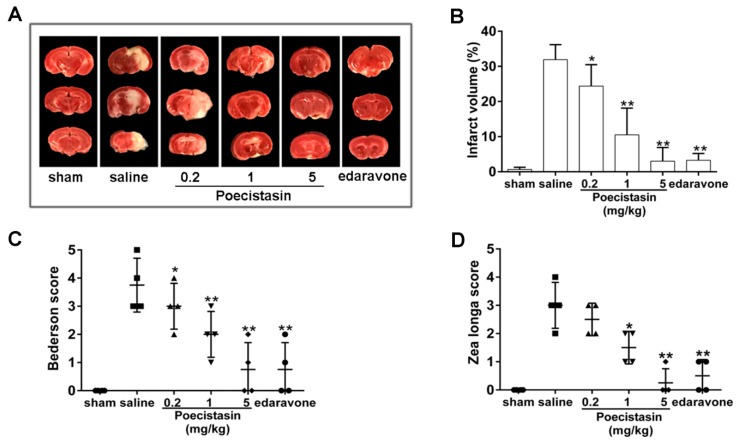
Effects of poecistasin on stroke model. (**A**) Representative coronal brain sections stained with 2, 3, 5-triphenyltetrazolium chloride (TTC) from mice on day 1 after tMCAO. The ischemic infarctions appear white. Brain infarct volumes (**B**) as measured by planimetry (% of the whole volume), Bederson score (**C**) and Zea longa score (**D**) in respective group on day 1 after tMCAO are also shown. Data represent mean ± SD (*n* = 6), ** *p* < 0.01, * *p* < 0.05 by unpaired *t*-test.

The effect of poecistasin on thrombosis was further investigated by using a transient middle cerebral artery occlusion (tMCAO) mouse model. As illustrated in Figure 5A,B, the cerebral infarct volume was ~32% in the control mice, while different concentrations of recombinant poecistasin (0.2, 1 and 5 mg/kg) decreased infarct volume to ~25, 10 and 2.5%. Edaravone decreased infarct volume to ~2.7%. The functional outcomes reflected by Bederson score and Zea longa score further indicated that poecistasin can alleviate ischemic stroke (IS) (Figure 5C,D). 

## 3. Discussion

Several antistasin-type serine protease inhibitors containing two domains have been found from leeches, including antistasin from *Haementeria officinalis* and ghilanten from *Haementeria ghilianii* [22,28]. Hirustasin and Bdellastasin containing only one domain were identified from *Hirudo medicinalis* [29,30]. Piguamerin and guamerin containing one domain was also identified from *Hirudo nipponia* [31,32]. In this new report, an antistasin-type serine protease inhibitor exerting anti-coagulatory effect named as poecistasin was firstly purified from leech *P. manillensis*. Poecistasin contains 48 amino acids and MALDI-TOF-MS analysis showed that the MW of native poecistasin was 5027.5 Da. Sequence of native poecistasin shows high similarity to antistasin-type serine protease inhibitors which contain five pairs of disulfide bonds forming with 1–3, 2–4, 5–8, 6–9 and 7–10 patterns [30] by ten cysteine residues. A guamerin-like homolog from *P. manillensis* [GenBank: ATE50007] that shares high sequence similarities to poecistasin (92% identical) has been reported. However, no functions and biological significances were studied. Enzyme activity test showed that poecistasin inhibited FXIIa, kallikrein, trypsin and elastase but it had no inhibitory effects on FXa and thrombin. The reactive P1 site was reported in previous works with leech antistasin-type inhibitors [30,31,32]. Antistasin, ghilanten, hirustasin, bdellastasin and piguamerin, which inhibit trypsin-like proteases, were reported to contain arginine or lysine at its predicted P1 site [30,31]. Guamerin, which inhibits chymotrypsin-like and elastase-like but not trypsin-like proteases, contains a methionine residue at its predicted P1 site [32]. Based on this works, we infer that putative poecistasin reactive P1 site locates at arginine 27. In accordance with an arginine being in P1 site, poecistasin is an inhibitor of trypsin. Different from other reported antistasin-type serine protease inhibitors from leeches, poecistasin inhibited both kallikrein and FXIIa of the intrinsic coagulation pathway, implying that poecistasin may influence intrinsic coagulation pathway. As expected, further research showed that poecistasin prolonged APTT in a dose-dependent manner but had no effect on PT. Blood-sucking animals get blood meal by overcoming host’s blood coagulation [34,35,36,37]. Actually, coagulation inhibitors have been found from *P. manillensis*, such as hirudin-like peptide homologs, hirudin is a kind of serine protease inhibitor exerts thrombin inhibitory activity [38]. Poecistasin may have potent function to help *P. manillensis* to take a blood meal by inhibiting coagulation at the bitten-site.

Mice carotid artery thrombosis induced by FeCl_3_ was used to evaluate the effect of poecistasin on thrombosis in vivo. Our results showed that poecistasin strongly inhibited blood flow and thrombus formation in a dose-dependent manner with a similar tendency to heparin sodium. The effect of poecistasin on thrombosis was further investigated by using a tMCAO mouse model and poecistasin alleviated infarct volume in dose-dependent manner and poecistasin showed stronger anti-IS activity than positive drug edaravone. These all imply poecistasin may be an excellent candidate for the development of clinical anti-thrombosis and anti-IS drugs. 

## 4. Conclusions

In conclusion, poecistasin was purified from *P. manillensis* secretions after three chromatographic steps-gel-filtration, RP-HPLC C8 and cation exchange, and its primary sequence identified by LC-MS/MS and MALDI-TOF analysis. Poecistasin can inhibit kallikrein and FXIIa of intrinsic coagulation pathway and proteases trypsin and elastase but has no inhibitory activity on FXa and thrombin under the same assay conditions. Interestingly, poecistasin was found to prolong APTT and had no influence on PT. Poecistasin showed anti-thrombosis activity with the similar effect of heparin sodium in FeCl_3_-induced carotid artery thrombosis model. Poecistasin also showed anti-IS effect with stronger activity than edaravone. Overall, poecistasin was the first antistasin-type serine protease inhibitor exerting anti-coagulatory effect from leech *P. manillensis* and might be an excellent candidate for the development of clinical anti-thrombosis and anti-IS drugs.

## 5. Materials and Methods 

### 5.1. Collection of Crude Secretions

*P. manillensis* leeches were purchased from Guangxi Province of China. The leeches were still alive when they were transported to the laboratory. Leeches were stimulated by an electrical stimulator and the crude secretions were collected. More specifically, leeches were stimulated by an electrical stimulator (6 volt, 2.5~345 Hz) around the mouth for 5~10 s, the secretions were washed for several times with stimulation buffer (150 mM NaCl, 1 mM L-Arginine). The secretions were centrifuged at 12,000× *g* for 1 h at 4 °C, and the supernatant was collected and stored at −80 °C.

### 5.2. Purification of Poecistasin

The supernatant of leech secretions was lyophilized, dissolved with 0.1 M PB buffer (Na_2_HPO_4_-NaH_2_PO_4_, pH 6.0) and separated by a Sephadex G-50 column (100 × 2.6 cm, GE Health, Chicago, IL, USA) that was previously equilibrated with the same buffer subsequently. By eluting the column with the same buffer, we got the sample fractions. The flow rate of eluting was 0.3 mL/min at 4 °C and fractions were collected once every 10 min. The absorbance of the elution fractions was monitored at both 215 and 280 nm. Fractions that inhibited FXIIa were pooled and lyophilized prior to further purification. The powder from the previous step was dissolved and loaded to RP-HPLC on a C_8_ column (30 × 0.46 cm, Hypersil BDS, Bellefonte, PA USA). Elution was carried out with a linear gradient of solution B (Acetonitrile, 0.1% TFA) at a flow rate of 0.7 mL/min and the absorbance of the elution fractions was monitored at 280 nm. The eluted fraction containing FXIIa inhibitory activity was collected. The eluted fraction of C_8_ RP-HPLC was lyophilized, resuspended with solvent A (20 mM MES, pH 6.0) and applied to a Mono S^TM^ 5/50 GL column (GE, Chicago, IL, USA) connected to AKTA explorer 10S FPLC system (GE, Chicago, IL, USA). The column was equilibrated with solvent A and the elution was performed with a linear gradient of 0–45% solvent B (20 mM MES, 1 M NaCl, pH 6.0) over 45 min at a flow rate of 1 mL/min and the absorbance of the elution fractions was monitored at both 215 and 280 nm. The fractions of previous step were also used to test FXIIa inhibitory activity. A lack of addition of any peak was used as negative control. 

### 5.3. Mass Spectrometric Analysis and Sequencing of Poecistasin

The eluted “peak 3” of FPLC exerting FXIIa inhibitory activity was collected and lyophilized. The MW of the collected peak was analyzed by MALDI-TOF-MS (AXIMA CFR, Kratos Analytical, Shimadzu Corporation, Kyoto, Japan). Peptide sequence was determined by liquid chromatography coupled with tandem mass spectrometry (LC−MS/MS) at Beijing Biotech-Pack Scientific Co., Ltd. (Beijing, China). Specifically, the purified peptide (peak 3, 100 μg) was transferred into Microcon devices YM-3 (Millipore, Billerica, MA, USA). The device was centrifuged at 12,000× *g* at 4 °C for 10 min. Subsequently, 200 μL of 50 mM ammonium bicarbonate were added to the concentrate followed by centrifugation and repeat once. After reduced by 10 mM DL-dithiothreitol (DTT) at 56 °C for 1 h and alkylated by 20 mM iodoacetamide (IAA) at room temperature in dark for 1h, the device was centrifuged at 12,000× *g* at 4 °C for 10 min and wash once with 50 mM ammonium bicarbonate. Added with 100 μL of 50 mM ammonium bicarbonate and free trypsin into the protein solution at a ratio of 1:50 and the solution was incubated at 37 °C overnight. The device was centrifuged at 12,000× *g* at 4 °C for 10 min. 100 μL of 50 mM ammonium bicarbonate was added into the device and centrifuged and then repeat once. Lyophilize the extracted peptides to near dryness. Resuspend peptides in 50 μL of 0.1% formic acid before LC-MS/MS analysis. Nanocolumn (100 μm × 10 cm) packed with a reversed-phase ReproSil-Pur C18-AQ resin (3 μm, 120 Å, Dr. Maisch GmbH, Ammerbuch, Germany) connected to Ultimate 3000 system (ThermoFisher Scientific, Waltham, MA, USA) combined with Orbitrap Elite™ Hybrid Ion Trap-Orbitrap Mass Spectrometer (Thermo Fisher Scientific, Waltham, MA, USA) was used to analyze the digestion product. The raw MS files were analyzed to deduce the peptide sequence.

### 5.4. Poecistasin Recombinant Expression and Purification

DNA encoding mature poecistasin was synthesized and the prokaryotic expression vector was constructed by inserting DNA sequence encoding mature poecistasin (144 bp) into pET-32a (+) vector (Novagen). Recombinant expression in *Escherichia coli* BL21 (DE3) was induced by 1 mM IPTG for 6 h in an 80-rpm shaker at 28 °C. Following expression, *E. coli* cells were collected by centrifuging at 12,000 rpm/min for 10 min at 4 °C and resuspended in binding buffer (20 mM Tris, 100 mM NaCl, pH 8.0) and then homogenized by using Ultrasonic Cell Disruption System (XINYI-IID, XinYi, China). Finally, the supernatant was collected by centrifuging for 1 h at 12,000 rpm/min at 4 °C. Ni^2+^ affinity chromatography column was equilibrated in advance with binding buffer. The collected supernatant containing fusion protein was subsequently loaded on a Ni^2+^ affinity chromatography column at the flow rate of 1 mL/min. The bound fusion proteins were eluted with 5 column volumes of elution buffer (20 mM Tris-HCl, 100 mM NaCl, 1 M imidazole, pH 8.0). The eluted fraction was resuspended in rTEV protease buffer (50 mM NaH_2_PO_4_, 150 mM NaCl) and the salt was removed by using Ultrafiltration device (Millipore, USA). rTEV protease (5 U/μL) was added into rTEV protease buffer containing poecistasin fusion proteins (1 mg/ml) and reacted for 14 h at 28 °C. The fraction from the previous step was loaded to RP-HPLC C_8_ column (30 × 0.46 cm) to purify recombinant poecistasin as the method described above. The purified recombinant poecistasin was applied to test FXIIa inhibitory activity. The MW of recombinant poecistasin was also analyzed by MALDI-TOF-MS.

### 5.5. Effects of Poecistasin on Proteases 

Effects of poecistasin on proteases including FXIIa, kallikrein, trypsin, elastase, FXa and thrombin were tested by using corresponding chromogenic substrates. The testing enzyme was incubated with different concentrations (0.5, 1 and 2 μM) of native poecistasin in 60 μL of 50 mM Tris buffer (pH 7.4) for 5 min and then a certain concentration of chromogenic substrate was added. The absorbance at 405 nm was monitored and the kinetic curve was recorded by using an enzyme-labeled instrument (Epoch BioTek, Winooski, VT, USA) for 10 min. Trypsin and elastase were all from Sigma and the enzyme concentrations used were 800 and 400 nM, respectively. The corresponding chromogenic substrates (Sigma, St. louis, MO, USA) were Gly-Arg-p-nitroanilide dihydrochloride for trypsin and N-Methoxysuccinyl-Ala-Ala-Pro-Val-p-nitroanilide for elastase, respectively. The concentration of all the substrates in the reactions was 0.2 mM. The concentrations of plasma kallikrein and FXa (Enzyme Research Laboratory, South Bend, IN, USA) were 80 and 20 nM, respectively, and the corresponding chromogenic substrates were H-D-Pro-Phe-Arg-pNA·2HCl (Hyphen Biomed, Neuville-sur-Oise, France) and CH_3_OCO-D-CHA-Gly-Arg-pNA-AcOH (Sigma, USA), respectively. The concentration of all the substrates in the reaction was 0.2 mM. The concentrations of human thrombin (Sigma, St. Louis, MO, USA) and FXIIa (Enzyme Research Laboratories, South Bend, IN, USA) were 10 nM and the corresponding chromogenic substrates were H-D-Phe-Pip-Arg-pNa·2HCl (0.2 mM, Hyphen Biomed, Neuville-sur-Oise, France) and H-D-Pro-Phe-Arg-pNA·2HCl (0.2 mM, Hyphen Biomed, Neuville-sur-Oise, France), respectively. A lack of addition of poecistasin was used as negative control. Dixon plot curve was used to calculate the *Kis* of poecistasin to inhibit the proteases as described [39].

### 5.6. APTT and PT Assays

Plasma from healthy human was collected from Kunming Blood Center. For APTT assay, 50 μL of APTT reagent (F008-1, Nanjing Jiancheng Bioengineering Institute, Nanjing, China) was incubated with 50 μL of plasma added with different concentrations of native poecistasin (0, 80, 120 and 160 nM). After 3 min incubation, 50 μL of CaCl_2_ (25 mM) preheated at 37 °C for 5 min was added and the clotting time was monitored at 405 nm using a semi-automatic coagulation analyzer (ThromboScreen 400c, Pacific hemostasis, Middletown, VA, USA). To test PT, 50 μL of plasma added with different concentrations of native poecistasin (0, 80, 120 and 160 nM) was incubated with 100 μL of PT reagent (F007, Nanjing Jiancheng Bioengineering Institute, Nanjing, China), which has been preheated at 37 °C for 15 min. The clotting time was then monitored at 405 nm. 

### 5.7. FeCl_3_-Induced Carotid Artery Injury Model

According to the method described [40], the mice (C57BL/6J, female, 23–25 g) were anesthetized and the body temperature was maintained at 37 °C throughout surgery. Different concentrations of recombinant poecistasin (0.2, 5, 10 mg/kg) and heparin sodium (20 mg/kg) were injected by tail vein 10 min before surgery. Saline was used as negative control. One of the carotid arteries was exposed by cervical incision and separated from the adherent tissue and vagus nerve. Thrombosis was induced by applying a piece (2 × 2 mm) of filter paper pre-soaked with 10% (*w*/*v*) FeCl_3_ solution to the exposed mice carotid artery. The blood flowing of the carotid artery of all groups were measured by the laser speckle perfusion imaging (PeriCam PSI, HR, Stockholm, Sweden) at 0, 5, 10, 15, 20, 25, and 30 min, respectively after FeCl_3_ induction. The perfusion unit of the region of interest (ROI) was also recorded. 

### 5.8. Stroke Model

The tMCAO model was applied to induce focal cerebral ischemia as the method described [41]. Mice (C57BL/6J, female, 23–25 g) were anesthetized with 2% isoflurane and tied on a heat controlled operating table (Harvard Apparatus, Holliston, MA, USA) to maintain at 37 °C during the whole period of surgery. Following a midline skin incision in the neck, the proximal common carotid artery and the external carotid artery (ECA) were ligated and a standardized silicon rubber-coated nylon monofilament (6023910PK10, Doccol, Sharon, Boston, MA, USA) was inserted and advanced via the right internal carotid artery to occlude the origin of the right middle cerebral artery. One hour later, mice were re-anesthetized, and the occluding filament was removed to allow reperfusion and different concentrations of recombinant poecistasin (0.2, 1 and 5 mg/kg) and edaravone (10 mg/kg) were injected by tail vein 10 min before reperfusion. For determining ischemic brain volume, mice were sacrificed after the induction of tMCAO for 24 h and the brain was quickly removed and cut into 2-mm thick coronal sections using a mouse brain slice matrix (Harvard Apparatus, Holliston, MA, USA). The brain sections were then stained with 2% TTC (T8877-5G, Sigma, St. Louis, MO, USA). The Bederson score and Zea longa score was tested to monitor neurological function. 

### 5.9. Animals and Ethics Statement 

All animal experiments were approved by the Animal Care and Use Committee at Kunming Institute of Zoology (identification code: SMKX-2016013; date of approval: 15 July 2016). Animal experiments conformed to the U.S. National Institutes of Health Guide for the Care and Use of Laboratory Animals (identification code: No. 85-23; date of approval: 2 January 1996). Mice (C57BL/6J, female, 23–25 g) were purchased from Vitalriver Experiment Animal Company (Beijing, China) and housed in a pathogen-free environment.

### 5.10. Statistical Analysis

For statistical analysis, the data obtained from independent experiments were presented as the mean ± SD. All statistical analyses were two-tailed and with 95% confidence intervals (CI) using GraphPad prism 6. The results were analyzed using an unpaired *t*-test. Differences were considered significant at *p* < 0.05.

## Supplementary Materials

The following are available online at http://www.mdpi.com/2072-6651/10/11/429/s1, Figure S1: Dixon plot curve to calculate the *Kis* of poecistasin to inhibit proteases; Figure S2: Recombinant expression and purification of poecistasin.

## References

[1] Birrell G.W., Earl S.T.H., Wallis T.P., Masci P.P., de Jersey J., Gorman J.J., Lavin M.F. (2007). The diversity of bioactive proteins in Australian snake venoms. Mol. Cell. Proteom..

[2] Ali M.F., Lips K.R., Knoop F.C., Fritzsch B., Miller C., Conlon J.M. (2002). Antimicrobial peptides and protease inhibitors in the skin secretions of the crawfish frog, Rana areolata. Bba-Proteins Proteom..

[3] Rawlings N.D., Barrett A.J., Bateman A. (2012). MEROPS: The database of proteolytic enzymes, their substrates and inhibitors. Nucleic Acids Res..

[4] Rawlings N.D., Barrett A.J., Bateman A. (2014). Using the MEROPS Database for Proteolytic Enzymes and Their Inhibitors and Substrates. Curr. Protoc. Bioinform..

[5] Bode W., Huber R. (1992). Natural Protein Proteinase-Inhibitors and Their Interaction with Proteinases. Eur. J. Biochem..

[6] Friedrich T., Kroger B., Bialojan S., Lemaire H.G., Hoffken H.W., Reuschenbach P., Otte M., Dodt J. (1993). A Kazal-Type Inhibitor with Thrombin Specificity from Rhodnius-Prolixus. J. Biol. Chem..

[7] Lu S.M., Lu W.Y., Qasim M.A., Anderson S., Apostol I., Ardelt W., Bigler T., Chiang Y.W., Cook J., James M.N.G. (2001). Predicting the reactivity of proteins from their sequence alone: Kazal family of protein inhibitors of serine proteinases. Proc. Natl. Acad. Sci. USA.

[8] Derache C., Epinette C., Roussel A., Gabant G., Cadene M., Korkmaz B., Gauthier F., Kellenberger C. (2012). Crystal structure of greglin, a novel non-classical Kazal inhibitor, in complex with subtilisin. FEBS J..

[9] Araujo M.S., Nunes V.A., Gozzo A.J., Sampaio M.U., Auerswald E., Ura N., Shimamoto K., Sampaio C.A.M. (1999). Preliminary characterization of a Kazal-type serine protease inhibitor from Caiman crocodilus yacare plasma. Immunopharmacology.

[10] Macedo M.L., Garcia V.A., Freire M., Richardson M. (2007). Characterization of a Kunitz trypsin inhibitor with a single disulfide bridge from seeds of Inga laurina (SW.) Willd. Phytochemistry.

[11] Marlor C.W., Delaria K.A., Davis G., Muller D.K., Greve J.M., Tamburini P.P. (1997). Identification and cloning of human placental bikunin, a novel serine protease inhibitor containing two Kunitz domains. J. Biol. Chem..

[12] Shimomura T., Denda K., Kitamura A., Kawaguchi T., Kito M., Kondo J., Kagaya S., Qin L., Takata H., Miyazawa K. (1997). Hepatocyte growth factor activator inhibitor, a novel Kunitz-type serine protease inhibitor. J. Biol. Chem..

[13] Birk Y. (1985). The Bowman-Birk Inhibitor—Trypsin-Inhibitor and Chymotrypsin-Inhibitor from Soybeans. Int. J. Pept. Protein Res..

[14] Kennedy A.R. (1998). The Bowman-Birk inhibitor from soybeans as an anticarcinogenic agent. Am. J. Clin. Nutr..

[15] Prakash B., Selvaraj S., Murthy M.R.N., Sreerama Y.N., Rao D.R., Gowda L.R. (1996). Analysis of the amino acid sequences of plant Bowman-Birk inhibitors. J. Mol. Evol..

[16] Inouye K., Tonomura B.I., Hiromi K. (2014). Interactions between Streptomyces Subtilisin Inhibitor (SSI) and α-Chymotrypsin. Agric. Biol. Chem..

[17] Taguchi S., Kojima S., Kumagai I., Ogawara H., Miura K., Momose H. (1992). Isolation and Partial Characterization of Ssi-Like Protease Inhibitors from Streptomyces. FEMS Microbiol. Lett..

[18] Taguchi S., Kikuchi H., Kojima S., Kumagai I., Nakase T., Miura K., Momose H. (1993). High-Frequency of Ssi-Like Protease Inhibitors among Streptomyces. Biosci. Biotechnol. Biochem..

[19] Kojima S., Fujimura K., Kumagai I., Miura K. (1994). Contribution of Salt Bridge in the Protease Inhibitor Ssi (Streptomyces Subtilisin Inhibitor) to Its Inhibitory-Action. FEBS Lett..

[20] Moreau T., Baranger K., Dade S., Dallet-Choisy S., Guyot N., Zani M.L. (2008). Multifaceted roles of human elafin and secretory leukocyte proteinase inhibitor (SLPI), two serine protease inhibitors of the chelonianin family. Biochimie.

[21] Francart C., Dauchez M., Alix A.J.P., Lippens G. (1997). Solution structure of r-elafin, a specific inhibitor of elastase. J. Mol. Biol..

[22] Tuszynski G.P., Gasic T.B., Gasic G.J. (1987). Isolation and Characterization of Antistasin—An Inhibitor of Metastasis and Coagulation. J. Biol. Chem..

[23] Dunwiddie C., Thornberry N.A., Bull H.G., Sardana M., Friedman P.A., Jacobs J.W., Simpson E. (1989). Antistasin, a Leech-Derived Inhibitor of Factor-Xa—Kinetic-Analysis of Enzyme-Inhibition and Identification of the Reactive Site. J. Biol. Chem..

[24] Theunissen H.J.M., Dijkema R., Swinkels J.C., Depoorter T.L., Vink P.M.F., Vondinther T.G. (1994). Mutational Analysis of Antistasin, an Inhibitor of Blood-Coagulation Factor-Xa Derived from the Mexican Leech Haementeria-Officinalis. Thromb. Res..

[25] Nikapitiya C., De Zoysa M., Oh C., Lee Y., Ekanayake P.M., Whang I., Choi C.Y., Lee J.S., Lee J. (2010). Disk abalone (Haliotis discus discus) expresses a novel antistasin-like serine protease inhibitor: Molecular cloning and immune response against bacterial infection. Fish Shellfish Immunol..

[26] Lee M., Tak E., Park S., Cho S., Hahn Y., Joo S., Lee D., Ahn C., Park S. (2010). Eisenstasin, new antistasin family inhibitor from the earthworm. Biologia.

[27] Przysiecki C.T., Joyce J.G., Keller P.M., Markus H.Z., Carty C.E., Hagopian A., Sardana M.K., Dunwiddie C.T., Ellis R.W., Miller W.J. (1992). Characterization of Recombinant Antistasin Secreted by Saccharomyces-Cerevisiae. Protein Expr. Purif..

[28] Blankenship D.T., Brankamp R.G., Manley G.D., Cardin A.D. (1990). Amino-Acid-Sequence of Ghilanten—Anticoagulant-Antimetastatic Principle of the South-American Leech, Haementeria-Ghilianii. Biochem. Biophys. Res. Commun..

[29] Sollner C., Mentele R., Eckerskorn C., Fritz H., Sommerhoff C.P. (1994). Isolation and Characterization of Hirustasin, an Antistasin-Type Serine-Proteinase Inhibitor from the Medical Leech Hirudo-Medicinalis. Eur. J. Biochem..

[30] Moser M., Auerswald E., Mentele R., Eckerskorn C., Fritz H., Fink E. (1998). Bdellastasin, a serine protease inhibitor of the antistasin family from the medical leech (Hirudo medicinalis)—Primary structure, expression in yeast, and characterisation of native and recombinant inhibitor. Eur. J. Biochem..

[31] Kim D.R., Kang K.W. (1998). Amino acid sequence of piguamerin, an antistasin-type protease inhibitor from the blood sucking leech Hirudo nipponia. Eur. J. Biochem..

[32] Jung H.I., Kim S.I., Ha K.S., Joe C.O., Kang K.W. (1995). Isolation and Characterization of Guamerin, a New Human-Leukocyte Elastase Inhibitor from Hirudo-Nipponia. J. Biol. Chem..

[33] Kim D.R., Hong S.J., Ha K.S., Joe C.O., Kang K.W. (1996). A cysteine-rich serine protease inhibitor (Guamerin II) from the non-blood sucking leech Whitmania edentula: Biochemical characterization and amino acid sequence analysis. J. Enzym. Inhibition Med. Chem..

[34] de Marco R., Lovato D.V., Torquato R.J., Clara R.O., Buarque D.S., Tanaka A.S. (2010). The first pacifastin elastase inhibitor characterized from a blood sucking animal. Peptides.

[35] Markwardt F. (2016). State-of-the-Art Review: Antithrombotic Agents from Hematophagous Animals. Clin. Appl. Thromb/Hemost..

[36] Mende K., Petoukhova O., Koulitchkova V., Schaub G.A., Lange U., Kaufmann R., Nowak G. (1999). Dipetalogastin, a potent thrombin inhibitor from the blood-sucking insect Dipetalogaster maximus—cDNA cloning, expression and characterization. Eur. J. Biochem..

[37] Dodt J. (1995). Anticoagulatory Substances of Bloodsucking Animals—From Hirudin to Hirudin Mimetics. Angew. Chem. Int. Ed..

[38] Nicastro G., Baumer L., Bolis G., Tato M. (1997). NMR solution structure of a novel hirudin variant HM2, N-terminal 1-47 and N64->V+G mutant. Biopolymers.

[39] Magalhaes A., Magalhaes H.P.B., Richardson M., Gontijo S., Ferreira R.N., Almeida A.P., Sanchez E.F. (2007). Purification and properties of a coagulant thrombin-like enzyme from the venom of Bothrops leucurus. Comp. Biochem. Physiol. Part A.

[40] Zhu W.F., Gregory J.C., Org E., Buffa J.A., Gupta N., Wang Z.N., Li L., Fu X.M., Wu Y.P., Mehrabian M. (2016). Gut Microbial Metabolite TMAO Enhances Platelet Hyperreactivity and Thrombosis Risk. Cell.

[41] Gob E., Reymann S., Langhauser F., Schuhmann M.K., Kraft P., Thielmann I., Gobel K., Brede M., Homola G., Solymosi L. (2015). Blocking of Plasma Kallikrein Ameliorates Stroke by Reducing Thromboinflammation. Ann. Neurol..

