# Supplementary material for: Isolation and Characterization of Poecistasin, an Anti-Thrombotic Antistasin-Type Serine Protease Inhibitor from Leech Poecilobdella manillensis
Source: Toxins (Basel). 2018 Oct 26;10(11):429. doi: 10.3390/toxins10110429 (PMC6265900; doi:10.3390/toxins10110429)
Supplement: Supplementary file 1 [file toxins-10-00429-s001.pdf]

Supplementary Materials: Isolation and Characterization of Poecistasin, an Anti-Thrombotic Antistasin-Type Serine Protease Inhibitor from Leech *Poecilobdella manillensis*

Xiaopeng Tang, Mengrou Chen, Zilei Duan, James Mwangi, Pengpeng Li and Ren Lai

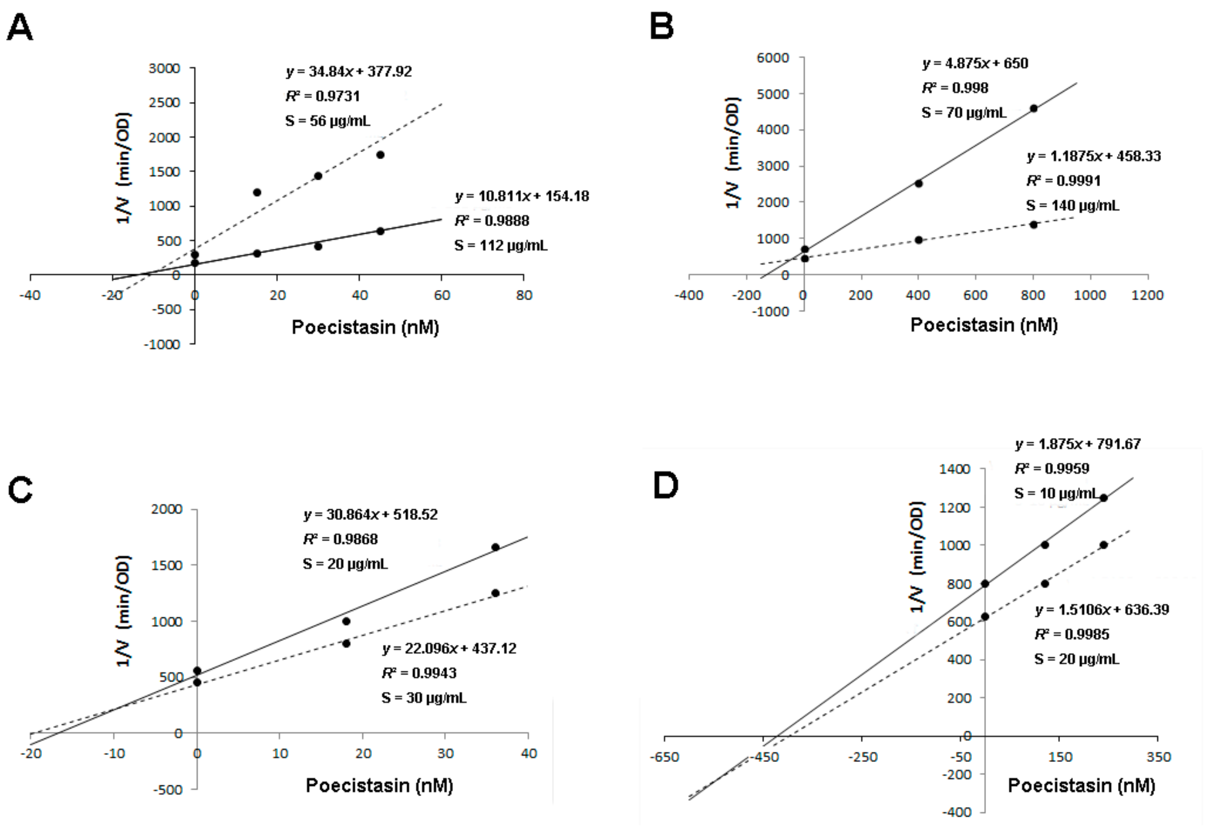

Figure S1. Dixon plot curve to calculate the Ki of poecistasin to inhibit proteases. Different concentrations of substrates for FXIIa, kallikrein, trypsin, and elastase were used to react with the corresponding enzymes,respectively. Dixon plot curve to calculate the Ki of poecistasin to inhibit FXIIa (**A**), kallikrein (**B**), trypsin (**C**), and elastase (**D**). The concentrations of substrate (S) and poecistasin were indicated in figure.

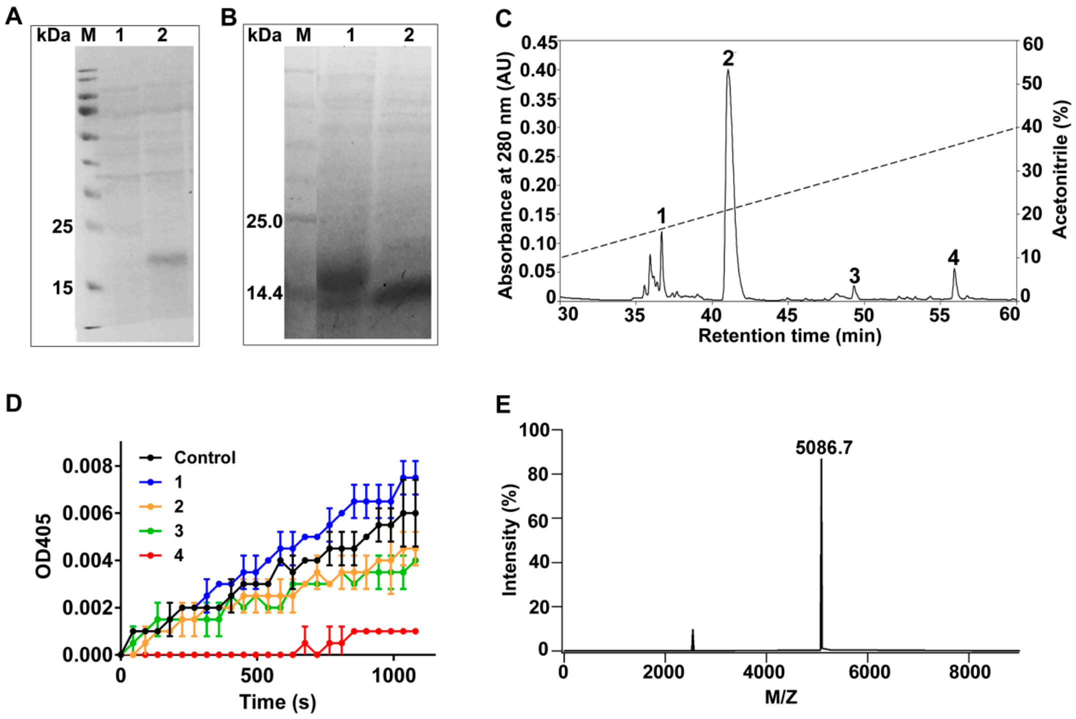

Figure S2. Recombinant expression and purification of poecistasin. (**A**) SDS-PAGE analysis of production of poecistasin fusion protein after the induction by 1 mM IPTG for 6 h in a 80-rpm shaker at 28°C. M, protein markers; lane 1, non-induced; lane 2, induced. (**B**) SDS-PAGE analysis of fraction of Ni^{2+} affinity chromatography column of supernatant fraction contained poecistasin fusion protein after cutting by rTEV protease (5 U/μL) in reaction buffer at 28°C for 14 h. M, protein marker; Lane 1, the supernatant fraction of Ni^{2+} affinity chromatography column; Lane 2, the fraction of "lane 1" after cutting by rTEV protease. (**C**) Purification of recombinant poecistasin by RP-HPLC C_8 column by monitoring at 280 nm. The dashed line represents a line gradient of acetonitrile from 10 to 40% over 30 min. (**D**) The peaks of "C" were used to test FXIIa inhibitory activity. (**E**) Mass spectrometry analysis of recombinant poecistasin (peak 4) by MALDI-TOF-MS.
